# Placebo Analgesia Does Not Reduce Empathy for Naturalistic Depictions of Others’ Pain in a Somatosensory Specific Way

**DOI:** 10.1093/texcom/tgab039

**Published:** 2021-06-02

**Authors:** Helena Hartmann, Federica Riva, Markus Rütgen, Claus Lamm

**Affiliations:** Social, Cognitive and Affective Neuroscience Unit, Department of Cognition, Emotion, and Methods in Psychology, Faculty of Psychology, University of Vienna, 1010 Vienna, Austria; Social, Cognitive and Affective Neuroscience Unit, Department of Cognition, Emotion, and Methods in Psychology, Faculty of Psychology, University of Vienna, 1010 Vienna, Austria; Social, Cognitive and Affective Neuroscience Unit, Department of Cognition, Emotion, and Methods in Psychology, Faculty of Psychology, University of Vienna, 1010 Vienna, Austria; Social, Cognitive and Affective Neuroscience Unit, Department of Cognition, Emotion, and Methods in Psychology, Faculty of Psychology, University of Vienna, 1010 Vienna, Austria

**Keywords:** empathy, fMRI, neuroscience, pain, picture-based, placebo analgesia, shared representations, social, somatosensation

## Abstract

The shared representations account postulates that sharing another’s pain recruits underlying brain functions also engaged during first-hand pain. Critically, direct causal evidence for this was mainly shown for affective pain processing, while the contribution of somatosensory processes to empathy remains controversial. This controversy may be explained, however, by experimental paradigms that did not direct attention towards a specific body part, or that did not employ naturalistic depictions of others’ pain. In this preregistered functional magnetic resonance imaging study, we aimed to test whether causal manipulation of first-hand pain affects empathy for naturalistic depictions of pain in a somatosensory-matched manner. Forty-five participants underwent a placebo analgesia induction in their right hand and observed pictures of other people’s right and left hands in pain. We found neither behavioral nor neural evidence for somatosensory-specific modulation of pain empathy. However, exploratory analyses revealed a general effect of the placebo on empathy, and higher brain activity in bilateral anterior insula when viewing others’ right hands in pain (i.e., corresponding to one’s own placebo hand). These results refine our knowledge regarding the neural mechanisms of pain empathy, and imply that the sharing of somatosensory representations seems to play less of a causal role than the one of affective representations.

## Introduction

Neuroimaging studies of empathy for pain have consistently found involvement of a network of brain regions related to affective-motivational processing of pain, such as anterior midcingulate cortices (aMCC) and anterior insula (AI). Other studies have highlighted the importance of sensory-discriminative processing in empathy, for example, in primary (S1) and secondary (S2) somatosensory cortices (Keysers et al. 2010 for a review; Fan et al. 2011; Timmers et al. 2018 for meta-analyses; Jauniaux et al. 2019; Fallon et al. 2020). Which regions are recruited depends strongly on the type of empathy task employed, in other words, whether the task focus lies on the “live” pain experience of another vs. on pictorial depictions of others’ pain (e.g., Lamm et al. 2011). Furthermore, the exact role of the somatosensory component during empathy remains controversially discussed. More specifically, it is unclear whether somatosensory pain processing networks contribute to empathy for pain by means of somatotopic matching, as they do for first-hand pain. This study thus had the following aims: First, and foremost, to investigate shared neural representations during pictorial depictions of others’ pain, and second, to resolve whether we specifically recruit somatosensory representations of our right hand, when empathizing with the pain of another person whose right hand is shown to be in pain on a picture. If this were the case, it would shed light on the putative role of shared somatosensory representations in empathy for pain, and extend our previous findings on effects of placebo analgesia on shared affective representations to the domain of picture-based presentations of others’ pain.

Empathy constitutes a complex, multifaceted phenomenon involving cognitive, affective, and behavioral processes and mechanisms (see e.g., Marsh 2018; Weisz and Zaki 2018 for recent reviews). Here, we refer to empathy as an affective state isomorphic to the affective state of another person that includes a partial and experiential sharing of the other’s affective state (de Vignemont and Singer 2006; Shamay-Tsoory and Lamm 2018; Hall and Schwartz 2019; Lamm et al. 2019). Interestingly, affective and somatosensory brain regions involved in processing empathic pain overlap in part with brain regions that are also active when we experience pain ourselves (Singer et al. 2004; Lamm et al. 2011). Within the so-called shared representations account of empathy, this activation overlap has been taken to suggest that shared representations may underlie such vicarious experiences. In other words, we seem to recruit the same neural substrates when feeling pain ourselves and when empathizing with someone else in pain (Bastiaansen et al. 2009; Bernhardt and Singer 2012). Although the majority of studies report this overlap of activation in aMCC and AI (Jackson et al. 2006; Corradi-Dell’Acqua et al. 2011; Benuzzi et al. 2018), other studies have pointed to S1 and/or S2 as equally relevant for pain empathy (Avenanti et al. 2005; Bufalari et al. 2007; Lamm et al. 2007a, 2007b; Novembre et al. 2015; Fabi and Leuthold 2017; Motoyama et al. 2017; Gallo et al. 2018; Riečanský and Lamm 2019 for a review). Evidence for shared representations was recently also confirmed with multivoxel pattern analyses (MVPA), which pinpointed the mid- to anterior-insular cortex and thus patches of cortex largely associated to affective processes as relevant for the sharing of self- and other-related pain representations (Zhou et al. 2020; but see also Krishnan et al. 2016). This raises the important question whether not only previously shown affective but also somatosensory representations of another’s pain experience are shared in pain empathy.

Importantly, an overlap in activation, as demonstrated, for example, with functional magnetic resonance imaging (fMRI), does not necessarily imply shared underlying functions and has fostered an active debate regarding the existence and location of shared representations underlying first-hand and empathy for pain. Trying to go beyond correlational evidence of shared activations towards evidence for shared representations, recent studies have made use of causal and/or psychopharmacological manipulations (Rütgen et al. 2015a, 2015b, 2018, 2021; Gallo et al. 2018). One such method is placebo analgesia, the reduction of first-hand pain by means of an inert pharmacological substance, which has been found to decrease both first-hand pain ratings and pain-related brain activity in regions such as insula, ACC, S1, S2, and thalamus (Wager et al. 2004; Eippert et al. 2009; Geuter et al. 2013; see Colloca et al. 2013; Atlas and Wager 2014 for a meta-analysis; Wager and Atlas 2015 for reviews). Moreover, placebo analgesia has been successfully employed to downregulate pain via localized manipulations, for example, using gels or creams on different body parts (Wager et al. 2004; Bingel et al. 2006; Geuter et al. 2013; Schenk et al. 2014; Schafer et al. 2015). Turning to empathy, Rütgen and colleagues used this method in three consecutive studies to test whether such a first-hand pain reduction transfers to empathy for pain, as the shared representations account would suggest (Rütgen et al. 2015a, 2015b, 2018). Indeed, global placebo analgesia by means of a placebo pill lowered activity in brain regions such as AI, dorsal ACC, S2, and thalamus, thus demonstrating that this induction procedure was indeed able to down-regulate first-hand somatosensory processing (Rütgen et al. 2015b). In the empathy condition, the authors further observed lower ratings of unpleasantness and empathy for pain as well as lower activity in aMCC and left AI in the placebo group compared to a control group who had not received any pill. They further showed a crucial role of the opioid system, as blocking placebo analgesia using the opioid antagonist naltrexone blocked the manipulation’s effects on first-hand and empathy for pain. A recent study from our lab showed additional domain-general effects, with placebo analgesia also affecting the first-hand and empathic experience of unpleasant touch, but pain-specific blockage of these effects using an opioid antagonist (Rütgen et al. 2021). The behavioral results for pain have since been replicated by an independent research group using real painkillers (Mischkowski et al. 2016) and extended to empathy for positive emotional states (Mischkowski et al. 2019). Similarly, hypnotic analgesia decreased brain responses of both self- and other-related pain in right AI and amygdala (Braboszcz et al. 2017).

Notwithstanding the important advances made by this line of research, several points are still unclear: First of all, these studies did not find any placebo-related modulation of the sensory-discriminative component of pain during empathy, suggesting that empathy for pain might be specifically focused on sharing the affective representation of another’s pain (Rütgen et al. 2015b). Relatedly, it has been proposed that specific types of empathy for pain paradigms recruit somatosensory brain regions to a larger degree, namely tasks where the attention is directed to the somatic location of pain, such as the exact body part in which the pain is inflicted (Lamm et al. 2011; Zaki et al. 2016; Xiang et al. 2018). Note in this context that two types of paradigms are commonly used in pain empathy research: cue-based tasks, which use abstract cues to indicate experimentally induced painful (electrical or thermal) stimulation in different intensities delivered to another person (usually on the hands or arms); and picture-based tasks, which show pictures or prerecorded videos of individuals’ body parts or faces in pain. Crucially, cue-based tasks usually employ an additional first-hand pain condition where the participants receive the same stimulation they are empathizing with (either in a separate block, or intermixed with the empathy condition), while picture-based tasks focus mostly on the empathic reaction to visual stimulus material (but see Corradi-Dell’Acqua et al. 2011; Krishnan et al. 2016; Zhou et al. 2020). One main conceptual difference between these paradigms is thus the way one infers the pain of another person, that is, either “live” versus via pictorial representations. Furthermore, in cue-based tasks, participants rely on implicit cues representing the target or intensity of the stimulation happening in that exact moment, while picture-based tasks show explicit and directly visible pain events through videos or pictures. Importantly, previous studies on placebo analgesia exclusively used cue-based setups with “live” experienced pain, and thus did not focus on depictions of others in painful situations (Rütgen et al. 2015a, 2015b). The present study aimed to close this gap by employing such a picture-based design. Most studies in the past employing picture-based tasks have not employed causal manipulations such as placebo analgesia. Only one line of research used transcranial magnetic stimulation to measure changes in corticospinal motor representations of hand muscles in individuals observing hands or feet being penetrated by needles (e.g., Avenanti et al. 2005; see Keysers et al. 2010 for a review). They showed a reduced amplitude of motor-evoked potentials in the muscles that participants saw being pricked by the needle, which correlated with subjective ratings of sensory pain qualities. This highlights the importance of somatic resonance and a direct, bodily matching of specific sensory aspects of others’ pain, leading to our second aim of investigating shared somatosensory representations with our picture-based setup.

We thus conducted a project employing a localized placebo analgesia manipulation on the right hand only (with the left hand acting as a control) and two paradigms tailored to investigate different yet related research questions. The first part of this project published elsewhere (Hartmann et al. 2021) aimed to refine the results of Rütgen et al. (2015b) regarding the attentional focus by using an adapted version of their cue-based task, where electrical stimulation was administered either to the participants or a confederate. In this setup, however, while increasing the focus on the affected body part, live pain to another person still had to be inferred via abstract cues indicating how painful stimulation of that body part was. In brief, this task did not reveal evidence for a location-specific somatosensory sharing of others’ pain. The second part of this project, reported here, aimed to investigate shared representations in a picture-based task consisting of naturalistic depictions of others in everyday painful situations. Beyond advancing our general understanding how placebo analgesia affects empathy in such a setup, this task also allowed us to focus on the role of lateralized somatosensory representations, as placebo analgesia had been induced only in the right hand, and the stimuli showed left and right hands. Our previous (Hartmann et al. 2021) and the present study thus used the same participant sample to investigate placebo-induced transfers in two distinct tasks that were conducted after each other in the same session.

In sum, it is currently unknown (1) whether shared representations also hold in picture-based empathy paradigms, and (2) whether pain empathy recruits first-hand somatosensory, body part-specific representations of that pain. The goal of the present, preregistered study was therefore to investigate whether locally applied placebo analgesia modulates the behavioral and neural responses to explicit naturalistic depictions of others in everyday painful situations. We tested this by experimentally reducing first-hand pain in one specific body part, the right hand, using a placebo gel. We hypothesized that behavioral and neural responses (both in affective and somatosensory brain regions) would be lower for stimuli corresponding to the right hand, where placebo analgesia was induced, compared with left/control hand stimuli. Our preregistered main predictions were, thus, that neural somatosensory representations engaged by first-hand pain are also recruited when empathizing with the pain of another person, and that the hand-specific reduction of first-hand pain would result in a corresponding hand-specific reduction of empathy for pain. If we indeed observed a location-specific reduction of pain empathy (lower pain ratings and lower brain activity in S1 and S2 for right hand- compared with left hand-related stimuli), this would imply shared representations at the level of somatosensation. If, on the other hand, we did not observe thisany kind of behavioral or neural differences between the left- and right-hand stimuli, it would suggest that somatosensory representations would play no specific role in empathy for pain. Such a finding would however be in line with previous reports on how placebo analgesia affects affective representations, which did not report modulation of somatosensory activity; as well as with our most recent study within the same sample, which did not find evidence for somatosensory modulation in a cue-based paradigm tailored to engage somatosensory areas.

## Materials and Methods

### Data and Code Availability Statement

Unthresholded statistical maps are available on NeuroVault (https://neurovault.org/collections/9244/). The stimuli of the picture-based empathy for pain task are available and will be shared individually upon request.

### Preregistration

In line with the suggestion for openly stating transparency by Simmons et al. (2012), we report how we determined our sample size, all data exclusions, all manipulations, and all measures in the study. This study was preregistered on the OSF prior to any creation of data (Hartmann et al. 2018; preregistration: osf.io/uwzb5; addendum: osf.io/h7v9p). The here reported part of the project was designed to extend the results of Rütgen et al. (2015b) regarding a transfer of first-hand placebo analgesia to empathy for everyday painful situations. The study design and procedures reported here are therefore largely identical to the ones employed in Hartmann et al. (2021), and reproduced here and in the Supplement Material. In the following methods and results, we clearly separate preregistered procedures and analyses from those added post hoc. Preregistered tests are additionally marked with a “p” in all results-related figures.

### Participants

For estimating the sample size, we conducted an a priori power analysis using GPower (Faul et al. 2007), using a conservative average of the lowest effect sizes from previous studies (one-sided paired *t*-test; see Rütgen et al. 2015a, 2015b). We aimed to detect a medium effect size of Cohen’s *d =* 0.40 at the standard 0.05 *α* error probability with a power of 1 – *ß* = 0.8, yielding a sample size of 41 participants. However, considering that the modulation of placebo analgesia might not be equal for empathy in everyday painful situations, 45 placebo responders were set as the data collection stopping-rule. Due to the nature of our research question, it was crucial to obtain a final set of participants who showed a first-hand placebo analgesia effect, in order to investigate a transfer of this effect to empathy for pain. We thus evaluated nonresponders to the placebo analgesia manipulation using a set of criteria consisting of three measures already employed in Rütgen et al. (2015b) and a fourth additional criterion possible due to our within-subjects design. In brief, those criteria were (1) strong doubts about the cover story, (2) low belief in the effectiveness of the “medication,” (3) a high number of conditioning trials, and (4) higher first-hand pain ratings for the right/placebo compared with the left/control hand in another task (see also sections M.1 in the Supplement Material for screening and exclusion criteria, and M.2 for nonresponder identification). From a total of 78 recruited participants, we excluded 20 (25.6%) nonresponders to the first-hand pain placebo manipulation, seven due to technical difficulties with the pain stimulator, five due to inconsistent ratings (e.g., equally high ratings for painful and nonpainful stimulation in both placebo and control conditions) and/or extensive movement and one participant because of a spontaneously found brain abnormality.

Our final sample included 22 males and 23 females between 19 and 32 years (*M* ± SD_age_ = 23.84 ± 2.73, all strongly right-handed with laterality quotients (LQs) ≥ 80 and normal or corrected-to-normal vision). Importantly, this sample of participants also conducted another task in the same session; the findings of this task are published in Hartmann et al. (2021). For the purpose of our study, we only recruited strongly right-handed participants and always induced the placebo in the right hand of all participants to avoid laterality-related problems in our fMRI analyses, increase sample homogeneity and retain comparability of the induction procedure. All participants gave written consent at each outset of their two sessions. Each participant received 50€ for taking part in both sessions and an amount aliquot to their time invested if they dropped out earlier. The study was approved by the ethics committee of the Medical University of Vienna (EK-Nr. 661/2011) and performed in line with the latest revision of the Declaration of Helsinki (2013).

### Procedure

Participants came to the MRI scanner, where the experimenter explained that the goal of the study was to investigate brain activity associated with a local anesthetic in the form of a medical gel. First, we performed an individual psychophysical pain calibration (a) to determine the maximum level of painful, but tolerable stimulation intensity and (b) to specify average subjective intensities on a visual analog scale (VAS) from 0 = not painful to 8 = extremely painful for very painful (rating of 7), medium painful (4) and not painful, but perceivable (1) stimulation for the left and right hand separately. Using the procedure employed by Rütgen et al. (2015b), electrical stimulation (stimulus duration = 500 ms) was administered to the dorsum of the hand using the Digitimer DS5 Isolated Bipolar Constant Current Stimulator (Digitimer Ltd, Clinical & Biomedical Research Instruments), one hand at a time, with two rounds going stepwise from very low (0.05 mA) to higher stimulation until the participant indicated the last given stimulus as an “extremely painful” (rating of 8). In a third round, stimuli with seemingly random intensity in the before calibrated range were delivered (although we alternated intensities corresponding to average ratings of 1, 4, and 7). A few seconds break between each stimulation and a few minutes break between each round ensured a reliable, independent rating of each stimulation.

The calibration was followed by the placebo analgesia induction. To this end, a medical student posing as the study doctor introduced the gel as a “powerful local anesthetic,” gave information on its effects and possible side effects, and then applied the placebo gel on the dorsum of the right hand. Participants were then told that a “control” gel without any active ingredients would be applied on the left hand. In fact, both the placebo and control gel contained nearly the same ingredients and no active pharmacological components (exact ingredients can be found in section M.3 in the Supplement Material). After 15 min of waiting time “for the medication to take effect,” we evaluated and amplified the effects of the placebo induction by means of a classic conditioning procedure. As in the calibration, participants were told that they would again receive electrical stimulation to the right or left hand via an attached electrode. Participants thought they would receive stimulation they had rated as very painful before on both hands. This was true for the left/control hand (average intensity rated as 7 during calibration); however, for the right/placebo hand, the experimenter secretly lowered the intensity to medium painful (rating of 4) to suggest pain relief. All participants completed a minimum of two and a maximum of four rounds and received oral feedback by the experimenter after each round. This was done to suggest that participant’s ratings on the left/control hand stayed similar to their average ratings during calibration, but the ratings on the right/placebo hand had decreased. This adjustment was done covertly, to maintain a participant’s belief that they received equally high intensities on either hand. Importantly, we purposefully chose a within-subject design and therefore did not directly test whether placebo analgesia leads to a general decrease in somatosensory regions, due to the absence of a control group not undergoing the placebo manipulation.

Afterwards, participants were led into the scanner room and, following general adjustments, completed two fMRI runs of another task (~45 min, findings reported in Hartmann et al. 2021), and one fMRI run of the picture-based empathy for pain task focused on here (~22 min) in a fixed order. Upon completion of all tasks, the field map and structural image were acquired. The session was concluded with postexperimental questionnaires and took around 4 h in total.

### Picture-Based Empathy for Pain Task

**Figure 1:**
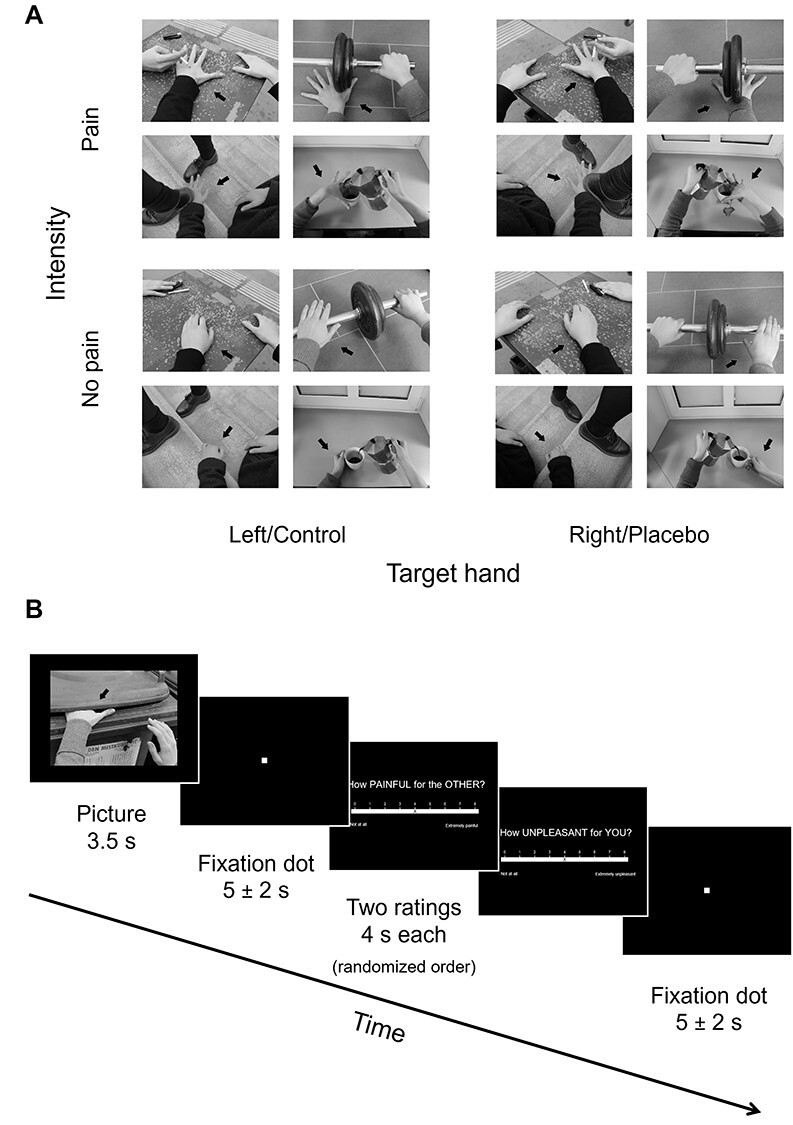
(*A*) Task stimuli showed everyday situations where one person (ostensibly accidentally) hurt himself/herself and varied depending on the target hand (left/control vs. right/placebo) and intensity (pain vs. no pain). Both hands were shown in all stimuli, but black arrows expressly indicated the hand to attend to and to rate in the trial. (*B*) Overview of the picture-based empathy for pain task. In a 2 x 2 within-subjects design, pictures depicted either painful or nonpainful everyday situations, and participants were asked to focus on either the left or the right hand (i.e., the one corresponding to their own control or placebo hand, respectively). The hand laterality to be attended to was marked again with a black arrow. Original stimuli in the task were colored.

To measure empathy for everyday painful situations, we adapted an established picture-based empathy for pain task from Jackson et al. (2005). Stimuli depicted everyday situations of one person accidently hurting himself/herself. For each situation, four pictures were taken, each picture always depicting both hands of a person taken from a first-person perspective (for the participant), but showing one out of four different conditions: left/control hand pain, right/placebo hand pain, left/control hand no pain, right/placebo hand no pain (see Fig. 1*A* for example stimuli and section M.4 in the Supplement Material for specific information regarding the stimuli creation and task presentation). A nonpreregistered, online validation study was conducted prior to the main study to select 15 situations out of 29 initial ones. To this end, we had recruited an independent sample of 38 right-handed participants (21 females, *M ±* SD_age_ = 30.50 ± 6.27). They were asked to rate 116 stimuli (29 situations x 2 intensities x 2 target hands) regarding other-related pain and one’s own unpleasantness when viewing the picture, both on 9-point visual analog scales from 0 = “not at all” to 8 = “extremely painful/unpleasant.” From the validation study, 15 out of the 29 situations rated as most painful were selected for the main task and evaluated on the following criteria: (i) the stimuli for left and right hand did not differ in the measured dimensions and (ii) the stimuli in the pain and no pain conditions did differ from each other. To ensure this, we performed two repeated-measures ANOVAs, for pain and unpleasantness ratings, each including the factors target hand (left vs. right hand) and intensity (pain vs. no pain) and the ratings from the 15 selected situations (see section M.4 and R.1 in the Supplement Material for description and results regarding the additionally assessed ratings of arousal, valence, and realism).

**Figure 2:**
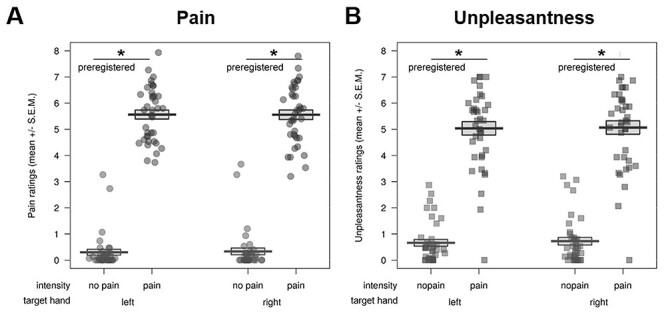
Preregistered behavioral results of the picture-based empathy for pain task, where participants rated pictures of others in painful and nonpainful everyday situations (displayed here as an index of the ratings for painful – nonpainful control stimuli). We observed no evidence for a transfer of the first-hand placebo effect induced using electrical pain to neither (*A*) empathy for pain nor (*B*) unpleasantness ratings, using a one-sided test of right/placebo hand < left/control hand. n.s. = not significant, SEM = standard error of the mean; BF_01_ = evidence for the null compared to the alternative hypothesis (also calculated one-sided); ^p^ = preregistered.

In the main task, 15 situations per condition were shown, resulting in 60 pictures/trials. The 60 images were included in one out of four pseudorandom trial orders previously created (see Fig. 1*B* for an overview of the trial structure in the task). Each trial consisted of the picture shown centered on a black background for 3500 ms, a jittered waiting period with a white fixation dot on black background for 5000 ± 2000 ms, two rating questions of 4000 ms each played in random order and a jittered intertrial-interval of 5000 ± 2000 ms, again with a white fixation dot on black background. The hand laterality that the participants had to rate was marked with a black arrow.

**Figure 3:**
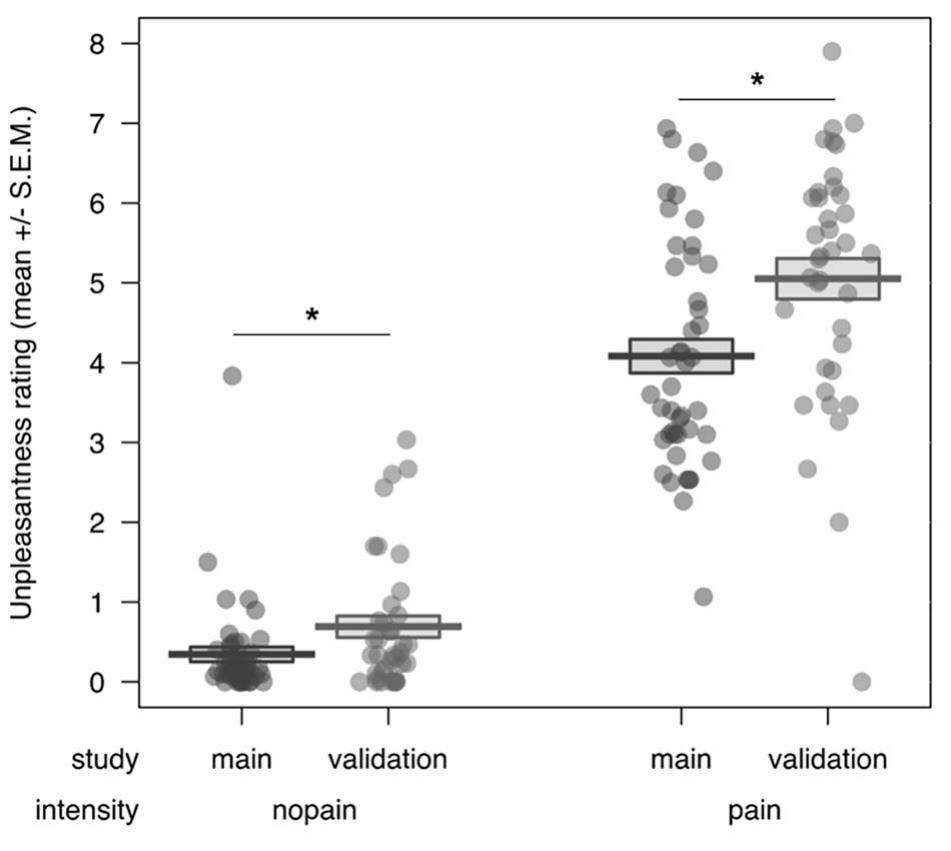
Posthoc comparison of unpleasantness rating to pictures of everyday painful situations between the validation and the main study (displayed here as an average of the ratings for stimuli depicting the right and left hand). We observed evidence for a significant, generalized reduction of unpleasantness in the main compared to the validation study, for pain as well as no pain stimuli. SEM = standard error of the mean.

Tapping into different aspects of empathy (Lamm and Majdandžić 2015), participants were asked to provide two ratings: (1) “How painful is it for the person in the picture?” and (2) “How unpleasant was it for you to view the picture?”. While the former question aimed to measure pain intensity, that is, the cognitive-evaluative aspect of the others pain, the latter question aimed to capture the affective-sharing aspect of the empathic experience. Questions were rated on the same 9-point scale from 0 = not perceivable to 8 = extremely painful/unpleasant.

### Data Acquisition

The empathy for pain task was implemented using the Cogent 2000 Toolbox Version 1.33 (http://www.vislab.ucl.ac.uk/cogent_2000.php) within MATLAB R2017b (Mathworks 2017). MRI data were acquired using a 3 Tesla Siemens Magnetom Skyra MRI-system with a 32-channel head coil (Siemens Medical, Erlangen, Germany). The functional scanning sequence included the following parameters: Echo time (TE)/repetition time (TR) = 34/1200 ms, flip angle = 66°, multiband acceleration factor = 4, interleaved ascending acquisition, interleaved multislice mode, matrix size = 96 × 96, field of view = 210 mm, voxel size = 2.2 × 2.2 × 2.0 mm^3^, 52 axial slices coplanar to the connecting line between anterior and posterior commissure, slice gap = 0.4 mm and slice thickness = 2 mm. Functional volumes were acquired in one run of ~22 min. Importantly, all participants completed three runs of fMRI data collection in total, with short breaks in between; the data reported here were always collected in the last run, while findings from the other two runs are reported in Hartmann et al. (2021). To acquire structural images, we used a magnetization-prepared rapid gradient-echo sequence (TE/TR = 2.43/2300 ms, flip angle = 8°, ascending acquisition, single shot multislice mode, field of view = 240 mm, voxel size = 0.8 × 0.8 × 0.8 mm^3^, 208 sagittal slices, slice thickness = 0.8 mm).

### Behavioral Data Analysis

All behavioral data were processed and statistically analyzed in RStudio Version 3.6.1 (R Core Team 2020; see section M.5 in the Supplement Material for information regarding analysis and plotting functions). Preregistered *t*-tests were conducted one-sided due to a priori, directional hypotheses. Cohen’s *d*’s for behavioral and fMRI analyses were calculated using the effect size calculation spreadsheet (version 4.2) provided by Lakens (2013).

#### Manipulation Checks

We conducted two manipulation checks to evaluate the strength of the first-hand placebo analgesia effect. First, we asked participants three times during the session how effective they believed the medication to be in reducing their own pain on the placebo-treated, right hand: after the gel application = preconditioning, after the conditioning = postconditioning and after all tasks in the postexperimental questionnaire = postsession. For this, we conducted three paired *t*-tests comparing the three time-points with each other. These analyses were preregistered as exploratory, but we expected an increase in belief between pre- and post-conditioning by the conditioning procedure as in Rütgen et al. (2015b).

Secondly, we used first-hand pain ratings from the two runs with the cue-based pain task preceding the picture-based task in the same imaging session. There, participants had received and rated electrical stimulation on either hand. As this measure (which was also one of the four preregistered criteria to identify nonresponders) was a reliable indicator of placebo analgesia, we also used it here as an additional manipulation check (see also section M.2 in the Supplement Material). To this end, we conducted a paired *t*-test using the first-hand pain ratings from that task (calculated as an index of pain – no pain). We further visually inspected the time course of the ratings related to the right/placebo hand to (a) pinpoint possible decreases of the placebo effect over the course of that task and (b) confirm that the placebo effect was intact and robust right before participants engaged in the picture-based task.

#### Preregistered Analyses

As those manipulation checks, and thus the induction of a first-hand placebo analgesia effect, were deemed successful, we conducted our main analyses to test whether the first-hand effect of placebo analgesia transferred to picture-based empathy for pain. In general, we employed a within-subject, full-factorial design with two factors of two levels each (target hand: left/control vs. right/placebo hand, intensity*:* pain vs. no pain). Two parametric two-factorial (2 x 2) repeated measures analyses of variance (ANOVAs) were used to analyze the results, where the dependent variables were either the pain or the unpleasantness ratings related to the viewing of the pictures. For each of the ANOVAs, we then compared the two hands on the index scores, separately for pain and unpleasantness ratings, using paired *t*-tests (always calculated as right/placebo hand (pain – no pain) < left/control hand (pain – no pain) and run one-tailed for rating_right/placebo hand_ < rating_left/control hand_).

#### Posthoc Analyses

As the pain-rating data were not normally distributed (indicated by a significant Shapiro–Wilk normality test), we additionally calculated a Wilcoxon rank-sign test which mirrored the results of our parametric test (see section R.2 in the Supplement Material). Because of the (absence of predicted) behavioral results, we added two Bayesian paired-samples *t*-tests to evaluate evidence of absence of effects (Keysers et al. 2020). These analyses mirrored the preregistered behavioral analyses, using pain and unpleasantness ratings separately as dependent variables and the default Cauchy (0, 0.707) prior of 0.707 as the effect size (this indicates a 50% chance to observe an effect size between −0.707 and 0.707; see e.g., Rouder et al. 2009). In general, a Bayesian *t*-test generates one Bayes Factor that compares relative evidence for the alternative versus null hypothesis (BF_10_, H_1_ vs. H_0_; BF_10_ ≤ 3: weak evidence, BF_10_ > 3: moderate evidence; BF_10_ > 30: strong evidence for H_1_) or the opposite, evidence for the null vs. the alternative hypothesis (BF_01_ = 1/BF_10_; BF_01_ ≤ 3: weak evidence, BF_01_ > 3: moderate evidence; BF_01_ > 30: strong evidence for H_0_) (Wagenmakers et al. 2011; Giolla and Ly 2019; van Doorn et al. 2020). Bayesian analyses were run one-tailed for rating_right/placebo hand_ < rating_left/control hand_ in JASP version 0.11.1 (JASP Team 2019).

The following section describes posthoc analyses we employed due to the null results found in the present study. As described below in the results, our present behavioral results showed no evidence for a placebo-induced decrease of subjective other-related pain or unpleasantness ratings related to the right/placebo hand. However, in another, previously published empathy task employing electrical stimulation conducted in the same participant sample and session, we did find evidence for a “generalized placebo effect”, that is, a general down-regulation of empathy for pain ratings in both left/control and right/placebo hand, independent of the localized placebo induction on only one hand (see also Supplement Material B of Hartmann et al. 2021). In other words, the placebo manipulation had a general effect on the rating of our stimuli, independent of the target hand. Therefore, we wanted to investigate the existence of such a generalized downregulation by comparing behavioral ratings of the pictures used in our main study to the same pictures used in the validation study, where no placebo manipulation was employed. This was done by calculating two between-subjects ANOVAs, including either the pain or the unpleasantness ratings in regard to the pictures, as well as the factors target hand (left/control vs. right/placebo hand), intensity (pain vs. no pain), and study (validation vs. main study). Effects indicating between-study differences were followed up using Welch’s *t*-tests and evaluated two-sided. Note that none of these analyses were preregistered as they resulted from pattern of results and additional insights and results originating between preregistration and data analysis, and should thus be considered as exploratory.

### fMRI Data Preprocessing and Analysis

#### Preprocessing and First-Level Analysis

Statistical Parametric Mapping (SPM12, Wellcome Trust Centre for Neuroimaging, https://www.fil.ion.ucl.ac.uk/spm/software/spm12/) running on MATLAB Version R2017b (Mathworks 2017) was used for preprocessing and statistically analyzing the MRI data. Preprocessing involved slice timing (reference = middle slice; Sladky et al. 2011), realignment with each participant’s individual field map, coregistration of structural and functional images, segmentation into gray matter, white matter (WM) and cerebrospinal fluid (CSF), spatial normalization, and spatial smoothing (8 mm full-width at half-maximum Gaussian kernel). The first-level design matrix of each participant contained four condition regressors and one for all ratings. For each condition, the onset and viewing duration (3.5 s) of the pictures were modeled as blocks and convolved with SPM12’s standard canonical hemodynamic response function. Six realignment parameters and two regressors modeling WM and CSF were included as nuisance regressors (WM and CSF values were extracted using the REX toolbox by Duff et al. 2007).

#### Preregistered Analyses

To test our hypothesis of a transfer of the first-hand localized placebo effect to empathy for everyday painful situations, we extracted and analyzed brain activation in three regions of interest (ROIs), determined independently from our data based on a meta-analysis on pain empathy (Lamm et al. 2011) and also used in our previous studies (Rütgen et al. 2015b; Hartmann et al. 2021): left AI (*x* = −40, *y* = 22, *z* = 0), right AI (39, 23, −4), and aMCC (−2, 23, 40). Additionally, we analyzed four ROIs in bilateral S1 (left S1: −39, −30, 51; right S1: 36, −36, 48) and S2 (left S2: −39, −15, 18; right S2: 39, −15, 18), taken from independent findings investigating somatosensory pain perception (Bingel et al. 2004, 2007). We created 10 mm spheres around each of the seven coordinates with MarsBaR (Brett et al. 2002) and extracted parameter estimates for each ROI using the first-level contrast images of each participant and condition with REX (Duff et al. 2007). ROI analyses were run in RStudio Version 3.6.1 (R Core Team 2020). As in the behavioral analysis, we employed the same within-subjects, full-factorial design including two factors (target hand, intensity) of two levels each, and the additional factor ROI with seven levels (lAI, rAI, aMCC, lS1, rS1, lS2, rS2). We first calculated an ANOVA pooling the activation of all seven ROIs and then calculated separate ANOVAs and planned comparisons for each ROI. Each ANOVA was followed up by a planned comparison between the two hands using an index of the activation (pain – no pain). Since Mauchly’s test for sphericity was statistically significant in the pooled ANOVA for the main effect of ROI and all interactions with the factor ROI, we reported the results of this ANOVA using Greenhouse Geisser sphericity correction. After observing a significant main effect of and interactions with the factor ROI in the initial three-way ANOVA, we went on with our preregistered plan and computed separate ANOVAs and planned comparisons for each ROI (one-sided tests for activity_right/placebo hand_ < activity_left/control hand_). To control for multiple comparisons, we corrected the calculated *t*-tests using Bonferroni correction by dividing the *α* error probability by the number of ROIs (for tests in affective ROIs: *P* = 0.05/3 ROIs = 0.017; somatosensory ROIs: *P* = 0.05/4 ROIs = 0.013).

#### Posthoc Analyses

Again, due to the (absence of predicted and exploratory evidence for opposite) results, we ran Bayesian paired-samples *t*-tests for each of the seven ROIs in JASP. The analyses of left and right S1 and S2 mirrored the preregistered fMRI analyses, and were run one-sided for activity_right/placebo hand_ < activity_left/control hand_ using the default Cauchy (0, 0.707) prior of 0.707 as the effect size. However, the analyses for the three affective ROIs (lAI, rAI, and aMCC) were run one-sided for activity_right/placebo hand_ > activity_left/control hand_ to investigate evidence for the opposite effect.

Furthermore, we explored hand-related differences in other regions apart from the ones preregistered by conducting an additional whole brain analysis for the contrasts activity_right/placebo hand_ < activity_left/control hand_ and activity_left/control hand_ < activity_right/placebo hand_, both calculated as pain – no pain (FWE-corrected at cluster level, *k* = 303).

## Results

### Behavioral Results

#### Validation Study

The aim of the validation study was to ensure that (i) the stimuli for left and right hand did not differ and that (ii) the stimuli in the pain and no pain conditions did differ from each other. We calculated five repeated measures ANOVAs for each of the five rating scales (pain, unpleasantness, realism, arousal, and valence) including the factors target hand (left vs. right hand) and intensity (pain vs. no pain). The results showed significant main effects of intensity in all ANOVAs (see Table S1 in the Supplement Material). Participants judged painful stimuli as significantly more painful (*F*(1,37) = 538.24, *P* < 0.001, *η*^2^ = 0.89; S1A in the Supplement Material) and unpleasant (*F*(1,37) = 253.65, *P* < 0.001, *η*^2^ = 0.75; S1B in the Supplement Material) as their nonpainful counterparts. However, we did not find any significant differences in our measured variables between the two hands (main effect of target hand) or any interaction between intensity and target hand. These results successfully demonstrated the validity of our created task stimuli.

#### Manipulation Checks

Next, we conducted two manipulation checks to evaluate the existence of a first-hand placebo analgesia effect. In the first check measuring beliefs in the effectiveness of the “medication” over the course of the session, means ± standard errors of the mean (SEM) were 6.64 ± 0.27 preconditioning, 8.06 ± 0.19 postconditioning and 6.71 ± 0.39 postsession. The increase in effectiveness beliefs as a result of the conditioning procedure was significant (pre- vs. postconditioning: *t*(44) = 5.91, *P* < 0.001, *M*_diff_ = 1.42, 95% CI_meandiff_ [0.94, 1.91], see S2A).

Participants’ beliefs dropped after the completion of the task (postconditioning vs. postsession: *t*(44) = −3.80, *P* < 0.001, *M*_diff_ = −1.36, 95% CI_meandiff_ [−2.07, −0.64]), but did not drop lower than the initial effectiveness belief after gel application after the whole session (preconditioning vs. postsession: *t*(44) = 0.16, *P* = 0.875, *M*_diff_ = 0.07, 95% CI_meandiff_ [−0.78, 0.92]).

In the second check, we found a significant difference between the participant’s placebo-treated versus control-treated hands for first-hand pain ratings in the task preceding the picture-based task, with a very high effect size (*t*(44) = 9.49, *P* < 0.001 one-sided, *M*_diff_ = 1.619, 95% CI_meandiff_ [1.28, 1.96], Cohen’s *d_z_* = 1.42; see S2B in the Supplementary Material). This difference was related to significantly lower pain ratings for the right hand (*M* ± SEM = 3.64 ± 0.21), that is, the hand in which placebo analgesia was induced, compared with the left, control-treated hand (*M* ± SEM = 5.26 ± 0.15). Importantly, visual inspection of those first-hand pain ratings showed no sign of decrease over time and posthoc analysis of this time course also did not reveal any significant effects of trial number (*P*’s > 0.281), suggesting a stable and robust placebo effect right before participants engaged in the picture-based task reported here.

In sum, the two manipulation checks showed (a) a strong belief in the effectiveness of the placebo gel that was highest right before entering the scanner, and (b) lower subjective, first-hand pain ratings in a task that was done right before the picture-based empathy task we focus on here.

#### Preregistered Analyses

Then, we went on to test our hypothesis and evaluate the existence of a transfer of the lateralized first-hand placebo analgesia effect to empathy for everyday painful situations in the main study. To this end, we calculated two repeated-measures ANOVAs (preregistered, see Tables S2 and S3 in the Supplement Material). The first repeated-measures ANOVA using the pain ratings of the pictures revealed main effects of target hand (*F*(1,44) = 5.42, *P* = 0.025 two-sided) and intensity (*F*(1,44) = 1348.88, *P* < 0.001 two-sided), indicating that there were significantly higher pain ratings for the left/control versus the right/placebo hand (*M*_right_ ± SEM = 3.02 ± 0.30; *M*_left_ ± SEM = 3.13 ± 0.30), and significantly higher pain ratings for painful versus nonpainful pictures (*M*_pain_ ± SEM = 5.78 ± 0.09; *M*_nopain_ ± SEM = 0.37 ± 0.07; see Fig. 2 for an overview of all ratings). However, we did not observe a target hand x intensity interaction (*P* = 0.711 two-sided), which would have shown evidence for pain-specific effects of the placebo manipulation. Paired comparisons mirrored those results, with no difference between the ratings (calculated as an index of painful – nonpainful stimuli) of left/control (*M* ± SEM = 5.38 ± 0.17) and right/placebo (*M* ± SEM = 5.42 ± 0.14) hands (*t*(44) = −0.37, *P* = 0.823 one-sided, *M*_diff_ = −0.037, 95% CI_meandiff_ [−0.24, 0.16], Cohen’s *d* = 0.03).

Results were similar in the second repeated-measures ANOVA using the unpleasantness ratings of the pictures. Here, we again observed main effects of target hand (*F*(1,44) = 15.56, *P* < 0.001 two-sided) and intensity (*F*(1,44) = 327.89, *P* < 0.001 two-sided), but opposite to the effect found in the pain ratings, there were significantly higher unpleasantness ratings for the right/placebo versus the left/control hand (*M*_right_ ± SEM = 2.29 ± 0.23; *M*_left_ ± SEM = 2.14 ± 0.23), and significantly higher unpleasantness ratings for painful versus nonpainful pictures (*M*_pain_ ± SEM = 4.08 ± 0.15; *M*_nopain_ ± SEM = 0.35 ± 0.07). Again, we observed no target hand x intensity interaction (*P* = 0.160 two-sided), which would again have shown evidence for pain-specific effects. Paired comparisons (using an index of the ratings for painful – nonpainful stimuli) showed no difference between the two hands (left: *M* ± SEM = 3.67 ± 0.22; right: 3.80 ± 0.21) for unpleasantness ratings (*t*(44) = −1.43, *P* = 0.960 one-sided, *M*_diff_ = −0.13, 95% CI_meandiff_ [−0.32, 0.05], Cohen’s *d* = 0.09).

#### Posthoc Analyses

Complementing the above results, the two post hoc one-sided Bayesian *t*-tests showed moderate to strong evidence for absence of the investigated effect, both for pain (BF_01_ = 8.07) and unpleasantness ratings (BF_01_ = 13.99) of the main study.

When comparing the main study’s results to the one of the validation study (which had not included a placebo analgesia induction) in order to investigate a generalized effect of this manipulation in the main study, we observed main effects of target hand (*F*(1,81) = 4.00, *P* < 0.048 two-sided) and intensity (*F*(1,81) = 1649.51, *P* < 0.001 two-sided), but no main effect or interactions with study (see Fig. 3; for the full ANOVAs, see Tables S4 and S5 in the Supplement Material). The main effect of target hand showed that pictures of the right hand (*M ±* SEM = 3.04 ± 0.22) were rated as more painful than pictures of the left hand (*M ±* SEM = 2.98 ± 0.22) independent of the intensity or study, and that painful pictures (*M ±* SEM = 5.68 ± 0.07) were rated as more painful than nonpainful pictures (*M ±* SEM = 0.35 ± 0.06) independent of the target hand or study.

Looking at the unpleasantness ratings, however, we observed main effects of study (*F*(1,81) = 11.37, *P* = 0.001 two-sided), target hand (*F*(1,81) = 11.78, *P* < 0.001 two-sided) and intensity (*F*(1,81) = 576.74, *P* < 0.001 two-sided). Again, the main effect of target hand showed that pictures of the right hand (*M ±* SEM = 2.57 ± 0.18) were rated as more unpleasant than pictures of the left hand (*M ±* SEM = 2.46 ± 0.18) independent of the intensity or study, and that painful pictures (*M ±* SEM = 4.53 ± 0.12) were rated as more unpleasant than nonpainful pictures (*M ±* SEM = 0.50 ± 0.06) independent of the target hand or study. Interestingly, the main effect of study showed that, in general, pictures were rated as inducing more self-related unpleasant affect in the validation study (*M ±* SEM = 2.87 ± 0.20) compared with the main study (*M ±* SEM = 2.21 ± 0.16). We therefore followed up on the main effect of study by comparing the unpleasantness ratings for the two hands for the two studies, separately for pain and no pain (see Figure). Here we observed that, for both intensities, the average unpleasantness rating for either hand in the pictures was significantly higher in the validation compared to the main study (pain: *P* = 0.004, *M*_val_ ± SEM = 5.05 ± 0.25, *M*_main_ *±* SEM = 4.08 ± 0.21; no pain: *P* = 0.038, *M*_val_ ± SEM = 0.69 ± 0.14, *M*_main_ ± SEM = 0.35 ± 0.09).

In sum, the behavioral results indicated no transfer of the placebo analgesia effect induced for first-hand electrical pain to empathy for pain or one’s own unpleasantness during everyday situations. In other words, participants rated the pain intensity of other people in everyday situations as well as their own unpleasant affect when viewing such pictures equally high and independent of the placebo induction on the right hand. These results were confirmed by the posthoc Bayesian analyses. Interestingly, exploratory analyses indicated a possible down-regulatory effect of the placebo induction on unpleasantness ratings to the pictures in general, independent of the rated hand.

### fMRI Results

#### Preregistered Analyses

After successfully inducing a first-hand placebo analgesia effect in our participants, we went on to test our preregistered main hypothesis for a transfer of this effect to empathy for everyday painful situations using a ROI approach. To this end, we extracted parameter estimates in three affective (bilateral AI, aMCC) and four somatosensory ROIs (bilateral S1 and S2). However, using our preregistered one-sided tests and hypothesizing a reduction of brain activation for pictures corresponding to the right/placebo hand, we did not find results confirming our hypotheses (see Table 1 for an overview of all planned comparisons).

**Table 1:** Main ROI analyses testing for placebo effects via one-sided paired *t*-tests

Brain region	*M* ± SEM		*t*(44)^p^	*P*_one-sided_	*d_z_*
	Left/control hand	Right/placebo hand			
Pooled ROIs	0.08 ± 0.04	0.16 ± 0.04	-1.76	0.958	0.26
Left AI	0.29 ± 0.07	0.50 ± 0.08	-2.99	0.995	0.43
Right AI	0.08 ± 0.06	0.26 ± 0.07	-2.54	0.993	0.38
aMCC	0.22 ± 0.07	0.36 ± 0.08	-1.91	0.937	0.28
Left S1	0.05 ± 0.05	0.09 ± 0.06	-0.63	0.733	0.09
Right S1	−0.05 ± 0.05	−0.05 ± 0.06	-0.02	0.506	<0.01
Left S2	0.005 ± 0.03	0.001 ± 0.02	0.15	0.558	0.02
Right S2	−0.03 ± 0.03	−0.05 ± 0.02	0.73	0.766	0.11

Note: Planned comparisons for the ROI analyses to evaluate the transfer of a first-hand placebo analgesia effect to empathy for everyday painful situations. For bilateral AI and aMCC, we observed effects that were opposite to our preregistered predictions; *P* values for activity_right/placebo hand_ < activity_left/control hand_ are displayed one-sided and interpreted using Bonferroni correction for multiple comparisons (*P* = 0.017 for affective ROIs and *P* = 0.013 for somatosensory ROIs); AI = anterior insula; aMCC = anterior midcingulate cortex; r/lS1 = right/left primary somatosensory cortex; r/l S2 = right/left secondary somatosensory cortex; *M* = mean; SEM = standard error of the mean; *t* (degrees of freedom); *d_z_* = Cohen’s *d*; ^p^ = preregistered.

The pooled ANOVA showed significant main effects of intensity and ROI, a trend for target hand x intensity (*P* = 0.086 two-sided) as well as all interactions involving the factor ROI (see Table S6 in the Supplement Material for the full ANOVA table). The planned comparison encompassing activation of all ROIs revealed a nonsignificant result (*P* = 0.958 one-sided, *M*_diff_ = −0.08, 95% CI_meandiff_ [−0.17, 0.01]), with no difference in activation for the right/placebo and the left/control hand. As preregistered, and due to a significant main effect of ROI as well as significant interactions with the factor ROI, we went on to calculate single ANOVAs and complementary *t*-tests for each ROI separately.

**Figure 4:**
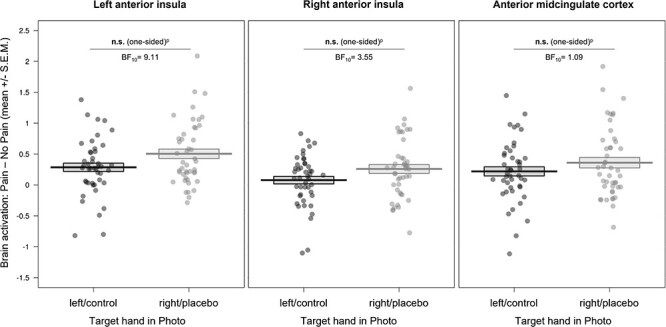
Affective ROI results of the empathy for pain task (displayed here as an index of activity related to painful – nonpainful control stimuli). We observed no evidence for a reduction of brain activity in affective regions related to empathy for pain by the placebo manipulation, using a one-sided test of right/placebo hand < left/control hand. Data inspection revealed the opposite effect, that is, higher activity in affective brain regions during empathy for pain in the placebo compared with the control condition. n.s. = not significant, SEM = standard error of the mean; BF_10_ = evidence for the alternative compared to the null hypothesis; ^p^ = preregistered.

Regarding the affective ROIs, we observed no evidence for lower placebo-induced hemodynamic activity in regard to pictures displaying the right/placebo compared to the left/control hand (left AI: *P* = 0.995 one-sided, *M*_diff_ = −0.22, 95% CI_meandiff_ [−0.37, −0.07]; right AI: *P* = 0.993 one-sided, *M*_diff_ = −0.18, 95% CI_meandiff_ [−0.32, −0.04]; aMCC: *P* = 0.937 one-sided, *M*_diff_ = −0.14, 95% CI_meandiff_ [−0.29, 0.01] also see Fig. 4 here and Tables S7–S9 in the Supplement Material). However, the two-sided ANOVAs and visual data inspection revealed evidence for an opposite effect (a target x hand interaction showing increased activity for the right/placebo compared with the left/control hand (lAI: *P* = 0.005; rAI: *P* = 0.015); see Tables S7 and S8 in the Supplement Material), which we followed up on using posthoc Bayesian analyses (see post-hoc analyses below).

**Figure 5:**
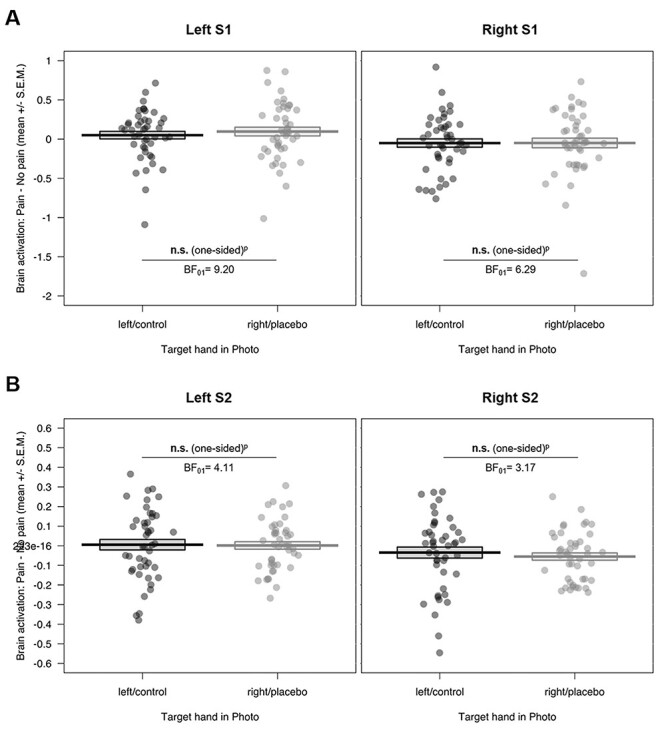
Somatosensory ROI results of the empathy for pain task for (*A*) bilateral S1 and (*B*) bilateral S2 (displayed here as an index of activity related to painful – nonpainful control stimuli). We observed no evidence for a reduction of brain activity in somatosensory regions related to empathy for pain by the placebo manipulation, using a one-sided test of right/placebo hand < left/control hand. n.s. = not significant, SEM = standard error of the mean; S1/S2 = primary/secondary somatosensory cortex; BF_01_ = evidence for the null compared to the alternative hypothesis; ^p^ = preregistered.

In the somatosensory ROIs, we again found no evidence for our preregistered hypotheses, as there were no differences in brain activation for the two target hands, neither in S1 nor in S2 (see Fig. 5 and Tables S10–S13 in the Supplement Material). More specifically, we did not observe the hypothesized lower brain activity related to right/placebo hand compared with left/control hand pictures (left S1: *P* = 0.733 one-sided, *M*_diff_ = −0.05, 95% CI_meandiff_ [−0.19, 0.10]; right S1: *P* = 0.506 one-sided, *M*_diff_ < −0.001, 95% CI_meandiff_ [−0.13, 0.13]; left S2: *P* = 0.558 one-sided, *M*_diff_ = 0.004, 95% CI_meandiff_ [−0.05, 0.06]; right S2: *P* = 0.766 one-sided, *M*_diff_ = 0.02, 95% CI_meandiff_ [−0.04, 0.08]).

#### Posthoc Analyses

As mentioned above, inspection of the data revealed an opposite pattern in bilateral AI and aMCC, whereby activation seemed to be higher for right/placebo hand compared with left/control hand pictures. This was underlined by the significant target hand x intensity interactions observed for bilateral AI (and a trend for aMCC) in our preregistered ANOVAs. Posthoc Bayesian analyses of the seven ROIs can be found in Table S14 in the Supplement Material. Importantly, those only showed moderate evidence for H_1_ compared to H_0_ for lAI (BF_10_ = 9.11) and rAI (BF_10_ = 3.55), that is, higher hemodynamic activity for right/placebo versus left/control hand pictures, but weak evidence for H1 in aMCC (BF_10_ = 1.09). In contrast, the Bayesian factors (BF_01_) for the somatosensory ROIs showed moderate evidence for the absence of a difference in activity.

The whole-brain analysis also did not reveal any evidence for other de- or increases in brain activity for right hand- compared with left hand-related stimuli, and vice versa.

In sum, the results, similar to the behavioral results, did not confirm our predictions of first-hand placebo analgesia reducing empathy for pain in both affective and somatosensory brain regions. However, exploratory posthoc analyses suggested an opposite effect, that is, higher hemodynamic activity related to right/placebo hand pictures in bilateral AI.

## Discussion

This preregistered study aimed to investigate the role of sensory-discriminative brain responses in empathy for everyday painful situations. To this end, we induced a localized placebo analgesia effect in the right hand of 45 participants using a placebo gel (with the left hand acting as a control) and then measured brain activity with fMRI during an empathy for pain task focusing on visual depictions of painful situations occurring to left or right hands.

Contrary to our preregistered predictions, we did not observe a localized reduction of self-reported empathy for pain or unpleasantness ratings related to the hand laterality as a result of the first-hand placebo analgesia induction. The neuroimaging results mirrored the behavioral findings, as we did not observe evidence for lower hemodynamic activity in any of our affective or somatosensory ROIs as a result of the placebo. These null results were bolstered by three additional findings: First, pain and unpleasantness ratings in the validation study showed a reliable differentiation of painful versus nonpainful stimuli, but no differences on those dimensions for pictures displaying the left versus right hand, speaking for the validity and comparability of our stimuli. Second, two initial manipulation checks showed an increase in the belief about medication effectiveness through the conditioning procedure, as well as a significant difference between first-hand pain ratings of the right/placebo and left/control hand in another task done in the same session (Hartmann et al. 2021). Together, these results demonstrated the successful induction of a localized, first-hand placebo analgesia effect, thus laying a strong foundation to investigate their possible transfer to the picture-based empathy task. Third, Bayesian analyses using the behavioral and brain data showed moderate to strong evidence for the absence of a transfer of lateralized placebo analgesia for first-hand pain to empathy for pain in our picture-based task. This suggests that shared somatosensory representations play less of a role during empathy for pain compared with shared affective representations, no matter how the pain of another person is inferred and where the attention of participants is directed. This interpretation is based on the observation that in two tasks both tailored to detect modulation of somatosensory sharing, we did not find evidence for modulation of such sharing by placebo analgesia. This contrasts with several previous studies that consistently reported modulation of processes and activity linked to affective representations (Rütgen et al. 2015a, 2015b, 2018, 2021).

Overall, our findings shed a different light on previous studies reporting an overlap of first-hand and empathy for pain in the sensory-discriminative component (Lamm et al. 2011 for a meta-analysis) and highlighting the role of somatosensory representations in empathy for pain (Avenanti et al. 2005; Bufalari et al. 2007; Riečanský and Lamm 2019). However, it is important to note that unlike before, our study specifically targeted somatotopic matching of self- and other-related pain states by using a localized placebo manipulation targeted only at the right hand. One possible explanation for the here reported absence of somatosensory sharing could be that the general processing of another’s pain might have a higher weight than processing the exact location and source of that pain, leading, in turn, to less involvement of the somatosensory network. Indeed, we cannot exclude a general down-regulation of somatosensory brain regions by placebo analgesia in the present study, as we did not have a between-subjects control group who was not under the influence of the placebo.

Our findings further tie in with studies investigating individuals with congenital insensitivity to pain (CIP), a patient group which is impaired in their first-hand and thus somatosensory pain processing (Danziger et al. 2006, 2009). CIP patients still show intact empathic responses despite this constraint, including the aMCC and AI engagement seen in neurotypical controls, as well as ventromedial prefrontal and posterior cingulate cortices. Interestingly, those results were found using mainly measurements of empathy for pain focusing on pictorial displays or visual imagery (i.e., images of body parts and facial expressions, video material of painful situations, and verbally presented imaginary painful situations).

We further report exploratory evidence for generally lower unpleasantness as a result of the placebo analgesia induction in the main study, independent of the displayed hands (compared to our prior validation study without such analgesia). Averaging over both hands, we demonstrated significantly higher unpleasantness ratings in the validation study, compared to the main study. This indicates that the placebo induction might indeed have had an effect on the participants, but not in the localized way we expected. Instead, unpleasantness ratings were generally lower in the main compared with the validation study independent of the hand laterality in the stimuli. This finding would be in line with our past work (Rütgen et al. 2015b) showing effects of a generalized placebo analgesia induction on affective brain networks, and with our previous study where we also observed such a general downregulation of empathic responses (Hartmann et al. 2021). More specifically, there we had observed, within the same sample of participants, a comparable reduction of empathy for pain ratings for both hands instead of the expected localized reduction for the right hand only. These results underline the conclusion that the placebo analgesia induction in the present project, encompassing the present study and the one published in Hartmann et al. (2021) may have influenced empathic resonance in a generalized, compared with a localized, way. Although this may not be too surprising, as the same subjects underwent this manipulation and did both tasks, it is interesting to note that this general effect was observed consistently over both tasks and is in line with other work from our lab reporting evidence for affective shared representations (Rütgen et al. 2015b). The absence of a localized transfer of the placebo effect raises the possibility of a domain-general affective blunting of empathy for pain responses in our study. However, we did not observe such a general blunting of responses during another task delivering first-hand electrical pain in the same session, where we saw a significant difference between the first-hand pain ratings of the two hands. Furthermore, a recent study from our lab (Rütgen et al. 2021) investigating exactly such domain-general effects showed that placebo analgesia does not transfer to pleasant touch, but only unpleasant touch and its empathic experience. In that study, only pain and empathy for pain, but not touch, were modulated by an opioidergic mechanism, which provided evidence for shared affective representations. Thus, it appears unlikely that our placebo induction procedure (which was very similar to the one in our previous studies, apart from employing placebo gel instead of pills) led to generally blunted affect but acted specifically on aspects related to the negative experience. Future studies should try to tease apart these specific versus general effects more thoroughly.

In terms of advancing our theoretical understanding of empathy and its neural underpinnings, the present findings extend the insights of our previous studies, which had provided evidence for shared opioidergic mechanisms between pain and empathy for pain. With the present project, we refined the apparent limits regarding the level of specificity when it comes to sharing others’ affective states. More specifically, it seems that we do not share others’ pain representations in a body part-specific manner. What seems to be shared in empathy for pain are the affective consequences of the painful situation, rather than their precise bodily origins.

In addition, we observed a main effect of hand in our preregistered analysis, that is, lower ratings for the right compared with left hand pictures independent of intensity, which could be another indication for a general placebo effect (especially as we did not observe this main effect in our validation study without any placebo induction). However, the actual difference in terms of rating was very small and previous between-subjects studies have reported an effect of the placebo only on pain (and not on no pain) ratings (Rütgen et al. 2015a, 2015b, 2018). Therefore, this reasoning about a general placebo effect is only preliminary and should be investigated further in future studies, especially because the validation and main study were not exactly the same: Apart from the 15 painful situations used in the validation and main study, the validation study employed a different set of participants, measured an additional 14 situations and also assessed realism, arousal and valence ratings for each picture. Although we chose the 15 most painful situations for the study comparison, the possibility of anchoring effects depending on the shown stimuli should be the subject of future research. In this context, an inherent limitation of our within-subjects design is the absence of a control condition for the placebo response, as all our participants underwent a placebo induction on their right hand. Although the validation study showed evidence for a generalized effect of the placebo, it can only supply indirect insights regarding the extent of placebo effects in the main study (see also Supplement Material B in Hartmann et al. 2021 for a more thorough discussion of this issue). Future studies should aim for including such a condition in order to draw more conclusive conclusions about a possible general placebo effect and shared affective representations on the brain level.

Furthermore, we found an unexpected opposite effect for bilateral AI, whereby hemodynamic activity was higher for pictures of the right hand (corresponding to the participant’s own placebo hand) compared with the left/control hand. It is important to note, though, that these explorations were not part of our preregistered hypotheses and have to be interpreted with caution. Critically, we did not preregister an analysis testing for this unexpected finding of higher brain activity for pictures relating to the right/placebo as opposed to the left/control hand, as we hypothesized the opposite effect and thus tested one-sided. This caveat, coupled with our null findings and the nature of the picture-based task, which did not allow comparison to the first-hand experience of the corresponding situations, limit the conclusions we can make in regard to somatosensory sharing of another’s pain. Although we report null results regarding our preregistered predictions, we briefly discuss our findings in the next section and highlight future research directions.

First, we induced the placebo only in the right hand of all our strongly right-handed participants, thus potentially confounding handedness with our placebo manipulation. It could therefore be that the right/placebo hand, always being the dominant hand, had specific effects on brain activation related to this hand, such as greater salience or attention. Indeed, Atlas and Wager (2014) discuss the possibility of placebo-induced changes in the insula and ACC changes reflecting attention or positive affective shifts. Future studies should therefore carefully test such laterality-related issues and extend this line of research, for example, by counterbalancing the placebo analgesia induction and/or adding left-handers to their sample.

Second, previous research regarding first-hand as well as empathy for pain has also highlighted the role of stimulus context and appraisal mechanisms (Lamm et al. 2007a, 2007b; Hein and Singer 2008 for a review; Atlas et al. 2010). For example, it has been shown that perspective changes can modulate evaluative processes and subsequent brain responses related to pain (Jackson et al. 2006), and that pain perception is shaped by multiple context factors such as expectations or beliefs (Jepma and Wager 2013; Jepma et al. 2018). In our task, for example, the context varied greatly between the pictures and each situation had to be interpreted differently, possibly requiring increased top–down perspective taking. In this line of reasoning, a failure of self-other distinction could have led to “empathic over-arousal” in the form of higher brain activation in AI in the placebo compared with the control condition. However, to avoid confusion of self- and other-related perspectives, but also mixing up of the hand laterality, we purposefully chose to display the pictures from an egocentric perspective and kept a black frame around the images.

Third, we carefully selected placebo responders by means of four specific criteria (Rütgen et al. 2015b; Hartmann et al. 2021), that is, only included participants showing a first-hand placebo analgesia effect. Wager et al. (2011) have highlighted a possibly altered process of pain evaluation in strong placebo responders, rather than a blockade of noxious input. Indeed, many previous studies not only find a decrease in pain-related brain regions but an additional increase in affective brain regions such as insula and rostral ACC for first-hand pain (Petrovic et al. 2002, 2005; Bingel et al. 2006), suggesting placebo-related modulatory mechanisms (also see Amanzio et al. 2013 and Atlas and Wager 2014 for meta-analyses and placebo-related increases in other regions). If the pain is evaluated differently to begin with, this could explain our paradoxical increase of brain activity in bilateral AI, corresponding to processing of right-hand pictures. However, this is only speculative and should be thoroughly tested in future studies, especially since the evidence observed here was only moderate and the majority of studies using placebo analgesia usually report a decrease of activity in insular regions.

Two other important points are task order and time effects. Although we took great care in inducing the placebo effect in a way that it was stable for the whole session, the task reported here was conducted after another task, around 45 min after the placebo induction was completed. This could have influenced the strength of the placebo effect and led to a decrease or general change over time. However, both of our manipulation checks showed a persistent effect until the end of the session, visible in comparable beliefs in the effectiveness of the gel from the start of the placebo induction to the end of the session, and in significantly lower (and stable) first-hand pain ratings for the right/placebo compared with the left/control hand in another task done right before the here presented empathy task. Furthermore, a previous study points to self-reinforcing expectancy effects in pain (Jepma et al. 2018). Indeed, the first-hand pain ratings in the placebo condition of the cue-based task completed directly before the picture-based task, showed no evidence for a decrease of the placebo effect over time.

As mentioned before, placebo analgesia in first-hand pain may have led not only to decreased brain activity in regions such as insula, ACC and S2, but also to increased activity in prefrontal regions such as dorsolateral, medial, and orbitofrontal cortices as well as inferior frontal gyrus (see e.g., Atlas and Wager 2014 for a meta-analysis). In line with this, neurons in the medial prefrontal areas might be involved in exerting placebo-related, modulatory control over an automatically triggered, affective response to the sight of highly aversive situations such as our stimuli (Lamm et al. 2007b). However, a complementary, exploratory whole brain analysis did not reveal any activation differences for right/placebo versus left/control hand in prefrontal areas in our task, when FWE-corrected at cluster-level. Investigating these brain regions in the context of analgesic effects on empathy for pain more specifically should therefore be the focus of future studies.

In conclusion, although our results suggest a successfully induced, localized placebo analgesia effect for first-hand pain, we did not find evidence for a somatotopically specific reduction in brain activation, when assessing empathy for everyday painful situations. This study suggests that shared somatosensory representations play less of a role during empathy for pain compared with previously reported shared affective representations. Our work thus refines how empathy is processed in our brains, and the extent and type of influence that our first-hand pain experience has on empathic responding.

## CRediT Author Statement

Helena Hartmann: Conceptualization, Data curation, Formal analysis, Funding acquisition, Investigation, Methodology, Software, Visualization, Writing—original draft, Writing—review and editing. Federica Riva: Formal analysis, Supervision, Writing—original draft, Writing—review and editing. Markus Rütgen: Formal analysis, Methodology, Supervision, Writing—original draft, Writing—review and editing. Claus Lamm: Conceptualization, Formal analysis, Funding acquisition, Methodology, Project administration, Resources, Supervision, Writing—original draft, Writing—review and editing (Brand et al. 2015).

## Supplementary Material

Manuscript_pimb18_pic_supplement_CCCfinal_tgab039

## Notes

We thank Ronald Sladky for valuable input on a final draft of the preregistration. We would also like to thank the two master students Anna Köstler and Fabian Franken who wrote their theses within this project as well as numerous interns and confederates for helping with the data collection. *Conflict of Interest*: H.H. works as a researcher and psychologist for MyMind GmbH, a company developing a neurofeedback training game for children with autism spectrum disorder, but this work is in no way related to the present research. The remaining authors declare that they have no financial interests or potential conflicts of interest.
