# Supplementary material for: Placebo Analgesia Does Not Reduce Empathy for Naturalistic Depictions of Others’ Pain in a Somatosensory Specific Way
Source: Cereb Cortex Commun. 2021 Jun 2;2(3):tgab039. doi: 10.1093/texcom/tgab039 (PMC8276832; doi:10.1093/texcom/tgab039)
Supplement: Manuscript_pimb18_pic_supplement_CCCfinal_tgab039 [file manuscript_pimb18_pic_supplement_cccfinal_tgab039.pdf]

Supplementary material

This study is part of a bigger project that has in part already been published, with the only difference being the task that was reported (Hartmann et al. 2021). Most of the information given here in M.1, M.2, M.3 and M.5 is therefore paraphrased from this previous paper.

Methods

M.1 Sample

Participants were recruited via a database of interested study participants, distributing flyers and advertising on social media. Interested participants first filled out an online questionnaire screening for any exclusion criteria (especially regarding MRI safety) and only eligible participants were invited to the study. Exclusion criteria were past/present enrollment in university studies including psychology, pharmaceutics or (human, veterinary, dental, etc.) medicine, any psychiatric or neurological conditions, past/present long term self-injurious behavior, past or present medical conditions regarding the hands that could possibly interfere with current pain sensitivity (e.g. chronic pain, numbness, trauma, injuries or long-term pain therapy), past/present alcohol or drug abuse, the intake of psychopharmacological medication in the last three months (besides oral contraceptives), left-handedness or no handedness preference, and a weakness in distinguishing left from right. The latter was operationalized by asking participants how often they mix up left and right in their daily life, e.g. while making a turn with the car. We excluded participants answering this question with ‘sometimes’, ‘often’ or ‘always’. Handedness was measured using a combination of two established handedness questionnaires (Oldfield 1971; Büsch et al. 2009). A laterality quotient (LQ; going from -100 = strongly left-handed to +100 = strongly right-handed) was calculated from the ten most selective items of the two questionnaires, and only individuals with LQs ≥ 72 were invited to the take part (Tran et al. 2014). Each participant was tested twice for any contraindications regarding MRI scanning, once via an online screening questionnaire and once in person at the outset of the scanning session. Excluded participants, such as dropouts and placebo analgesia nonresponders (see also next section S.2), were replaced until the preregistered sample size of 45 participants was reached.

M.2 Nonresponder identification

Placebo analgesia nonresponders were determined using four exclusion criteria (the first three criteria taken from (Rütgen et al. 2015): (1) We recorded any strong, verbally expressed doubts regarding the study setup during and after the session; (2) We collected belief scores about the effectiveness of the gel to decrease the participant's own pain at three time-points during the session (after the gel application = pre-conditioning, after the conditioning = post-conditioning, and in the post-experimental questionnaire = post-session, each rating on a continuous visual-analogue-scale from 0-10 cm). Pre- and post-conditioning ratings that were < 6.66 cm in sum (indicating a general low belief) and/or pre- minus post-conditioning ratings that were > 3.33 cm (indicating a decrease in belief after the conditioning) classified a participant as a nonresponder. (3) We counted the number of conditioning trials needed to show an analgesic response, with four or more trials indicating nonresponding. However, none of the participants needed more than three conditioning trials to show a response to the placebo; (4) During the data collection, we preregistered a fourth measure we had previously overlooked, as it had not been possible in the previous study (which mainly informed the pre-registration) due to a between-subjects design. This involved a first-hand pain task, where we applied short-lasting painful and non-painful electrical stimulation delivered to the right/placebo and left/control hands of the participant, and then collected pain ratings (for a more thorough report see also Hartmann et al. 2021). Using this fourth criterion, we could directly compare the self-related pain ratings of the two hands and excluded participants who showed higher average first-hand pain on the right/placebo compared to the left/control hand. This allowed us to identify responders with increased certainty, and thus maximize the placebo responsiveness of the sample as well as bolster interpretability of our results. During preregistration of this addendum, the so far collected data had not been observed or analyzed yet.

M.3 Procedure

In brief, the study consisted of two parts: First, participants were invited to an initial one-hour session to the Faculty of Psychology and were asked to fill out different trait personality

questionnaires on a computer for around one hour. For the second session (average interval of 32.86 ± 29.16 (*M* ± *SD*) days), participants were asked to refrain from alcohol, drugs or medication-intake 24 hours before the testing session as well as from intake of food or any other drink besides water one hour before.

In general, participants were asked to rate each stimulation as intuitively but also as accurately as possible. We calibrated the left and right hand individually for each participant (alternating the hand being calibrated first across participants), because previous studies found pain tolerances to vary depending on the hand laterality and dominance (Murray & Safferstone 1970; Pud et al. 2009).

For the placebo induction, a combination of verbal suggestions and classical conditioning techniques was employed. First, a medical student posing as the study doctor (either male or female) did a brief medical screening including a (pseudo) drug-test to increase participant's belief in the cover story that a real medication would be administered. The placebo gel was presented as a "potent, local anesthetic" with pain-reducing effects on the part of the skin where it is applied to for around 2-3 hours and that its maximum effectiveness would thus last the whole scanning time. Importantly, participants were told that the medical gel blocked pain receptors in a pharmacological way and would thus exert no effects on normal touch sensitivity or non-painful stimulation intensity. This was done to ensure a belief decrease due to an absence of possibly expected skin numbing. In addition, the "medication" was described as already being well established since many years, legally approved, and routinely used in e.g. dental procedures and chronic pain patients. Participants were told that possible side effects in very rare cases could be dry skin and slight skin irritation. The placebo gel was always applied first on the dorsum of the right hand and directly after that the control gel on the left hand, which was described as a normal basic skin cream and representing a basis for the participant's typical pain perception (the word "placebo" was never mentioned to the participants over the course of the experiment). Participants were told that the application of different gels on each hand was routinely done to ensure equal conditions and make them comparable for later analysis. Both the placebo and control gel

contained 0.5 g Carbomer, 0.09 g TRIS, 15 g undiluted Isopropanol, 10 g Propylenglykol, 3 g Glycerol and Aqua pur. ad 100 g, the only difference being 10 g (out of 100 g) Isopropanol in the placebo gel and 10 g basic skin cream ('Ultrasicc') instead of the Isopropanol in the control gel. This difference led to a clearly recognizable visual and olfactory distinction between the gels, while still keeping them matched with regard to tactile feeling and hydrating properties. Here we adhered to previously used procedures inducing placebo analgesia by means of topical creams and gels (Benedetti et al. 1999; Bingel et al. 2006; Geuter et al. 2013; Schenk et al. 2014). After the gel application on both hands, the participant was led outside the control room to (ostensibly) wait for the medication to take effect, and received detailed instructions regarding the task. Then we asked participants back in the control room and told them that a "pain test" would be used to check the effectiveness of the medication. Before the conditioning and application of new electrodes in the same location as during calibration, excess gel was removed, and the hands were disinfected with 70% isopropyl rubbing alcohol.

A conditioning round was deemed successful, if all stimuli on the right/placebo hand were rated with lower than 6 and all stimuli on the left/control hand higher than 5. After unsuccessful rounds, stimulation intensity was slightly adjusted, i.e., increased for the left/control hand and/or decreased for the right/placebo hand, in order to increase the contrast between the hands. In the first conditioning round three stimulations were administered, in subsequent rounds four.

M.4 Picture-based empathy for pain task

The pictures were taken with a Canon EOS 1100D Camera (ISO 6400, 12.2 Megapixel) from an egocentric perspective to facilitate "putting oneself in the shoes of the other", as no mental rotation of the hand position was required of the participants. As in the study by Jackson and colleagues (Jackson et al. 2005), all situations depicted events happening in everyday life, such as jamming the hand in a cupboard or burning your hand in the oven and used different types of pain (mechanical, thermal and pressure). Minor clean-up, image improvements and insertion of black arrows were done with Adobe Photoshop CS3. All

pictures were rescaled to 700x525 pixels for the task and displayed on a BOLD screen 32 LCD for fMRI (https://www.crsltd.com/tools-for-functional-imaging/mr-safe-displays/boldscreen-32-lcd-for-fmri/nest/boldscreen-32-technical-specification#npm) which participants watched through a mirror mounted on the head coil. The hand participants rated with was counterbalanced between but kept constant within participants over all tasks in order to avoid any effects in the right/placebo hand being related to hemodynamic activity during rating.

In the validation study, we additionally asked participants to judge the realism ("Can you imagine this happening to you in real life?") on a 9-point visual analogue scale from 0 = "not at all" to 8 = "extremely realistic", as well as arousal and valence of each picture (via 9-point Self-Assessment Manikins (SAM) from "calming" to "exciting" (arousal) and from "negative" to "positive" (valence); see e.g. Bynion and Feldner 2017).

M.5 Data analysis and plotting

The following packages (functions) were used for analyses and plotting in RStudio: plyr (ddply), dplyr (arrange), stats (shapiro.test, t.test, cor.test, aggregate), tidyr (gather, spread), reshape2 (dcast), ez (ezANOVA), yarrr (pirateplot) and ggplot2 (ggplot). All Figures were created in Microsoft PowerPoint.

Results

R.1 Validation study

In Table S1 and Figure S1, we report the ANOVA results of the validation study. Non-painful stimuli were rated as significantly more calming ($F(1,37) = 130.97$, $p < .001$, $\eta^2 = 0.53$) and positive ($F(1,37) = 109.92$, $p < .001$, $\eta^2 = 0.66$) than painful stimuli. Although stimuli were judged as realistic in general, painful stimuli were rated as significantly less realistic as non-painful stimuli ($F(1,37) = 83.77$, $p < .001$, $\eta^2 = 0.33$).

Importantly, although the validation study showed that stimuli were judged as realistic in general, painful stimuli were rated as significantly less realistic than non-painful stimuli. This is no surprise, as we asked how realistic participants imagine that the situations happen to them in everyday life and non-painful situations occur more often. Crucially, we did not find

any significant differences in pain, unpleasantness, realism, arousal or valence between pictures focusing on the right or left hand, showing that any possible differences in realism affected the processing of pictures related to either hand in a similar way.

Table S1
Behavioral results of the ANOVAs in the validation study.

Tests and effects	$F_{(1,37)}$	$p_{(two\text{-}tailed)}$	*gen.* η^2
**Pain**			
target hand	0.12	.736	< 0.001
intensity	538.24	< .001	0.89
target hand x intensity	0.41	.524	0.001
**Unpleasantness**			
target hand	1.21	.278	0.004
intensity	253.65	< .001	0.75
target hand x intensity	0.15	.701	< 0.001
**Arousal**			
target hand	0.54	.466	< 0.001
intensity	130.97	< .001	0.53
target hand x intensity	1.59	.215	0.001
**Valence**			
target hand	2.28	.139	< 0.001
intensity	109.92	< .001	0.66
target hand x intensity	1.55	.221	< 0.001
**Realism**			
target hand	1.99	.166	< 0.001
intensity	83.77	< .001	0.33
target hand x intensity	0.49	.490	< 0.001

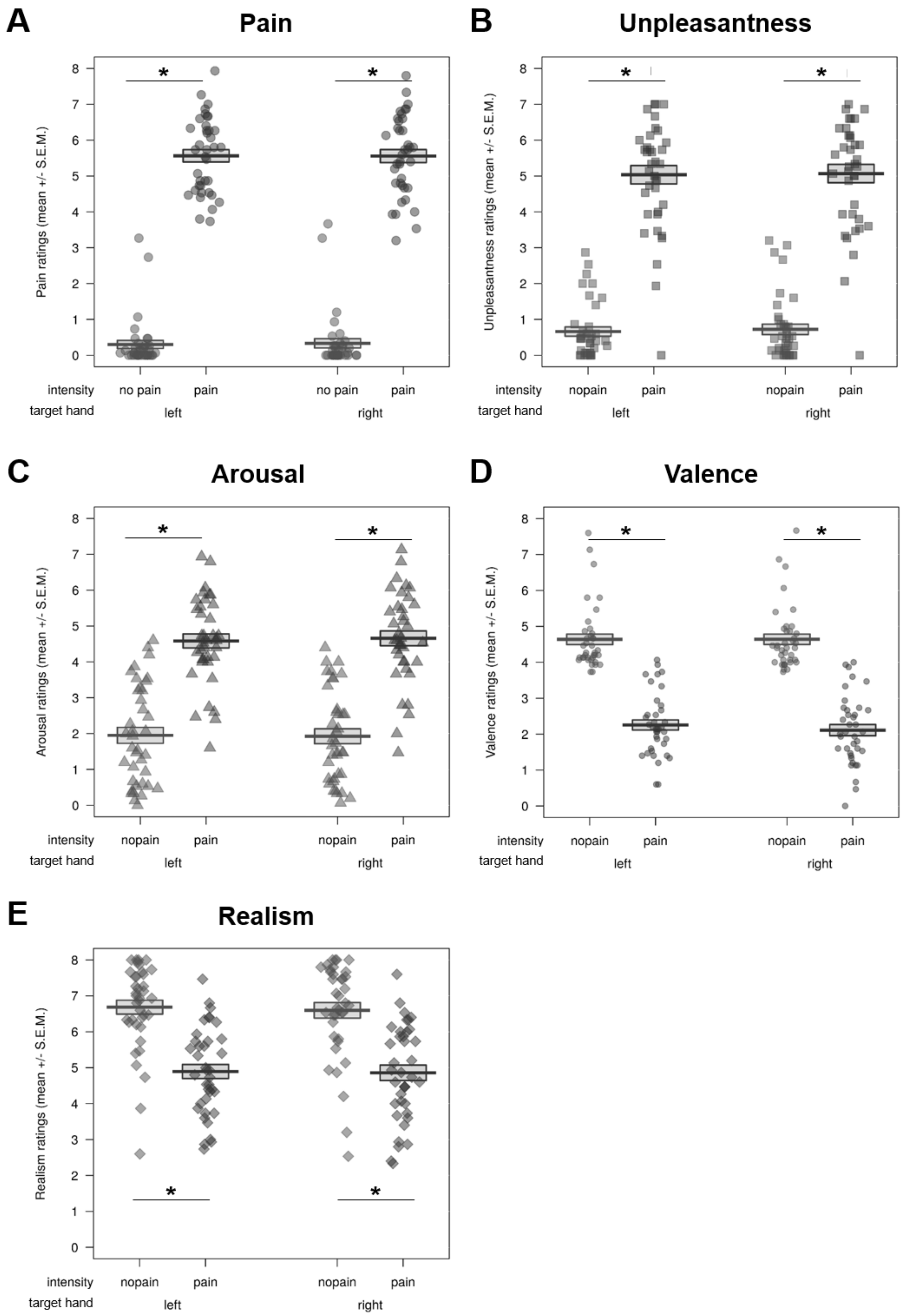

Figure S1. Behavioral results of the validation study. Participants rated picture stimuli of everyday painful and non-painful situations, separately for the left and right hand, and according to the following dimensions, all on 9-point Likert scales: A) pain (0 = “not at all” to 8 = “extremely painful”), B) unpleasantness (0 = “not at all” to 8 = “extremely unpleasant”). Apart from pain and unpleasantness, participants rated pictures of everyday painful/non-painful situations on three additional dimensions,

separately for the left and right hand, all on 9-point Likert scales: C) arousal (0 = “calming” to 8 = “exciting”), D) valence (0 = “negative” to 8 = “positive”) and E) realism (0 = “not at all” to 8 = “extremely realistic”). In all 2x2 ANOVAs using the factors target hand (left vs. right) and intensity (pain vs. no pain), we observed significant main effects of intensity, demonstrating that painful stimuli were rated as significantly more painful, more unpleasant, less realistic, more arousing and more negative than their non-painful counterparts. We found no significant differences in our variables between the two hands (main effect of target hand) or any interaction between intensity and target hand. The validation study was not preregistered, but conducted before (the preregistration of) the main study.

R.2 Manipulation checks

Here we report a figure of the manipulation check data, belief ratings about the effectiveness of the “medication” over the course of the session (Figure S2A) and first-hand electrical pain ratings in the task preceding the picture-based task (Figure S2B).

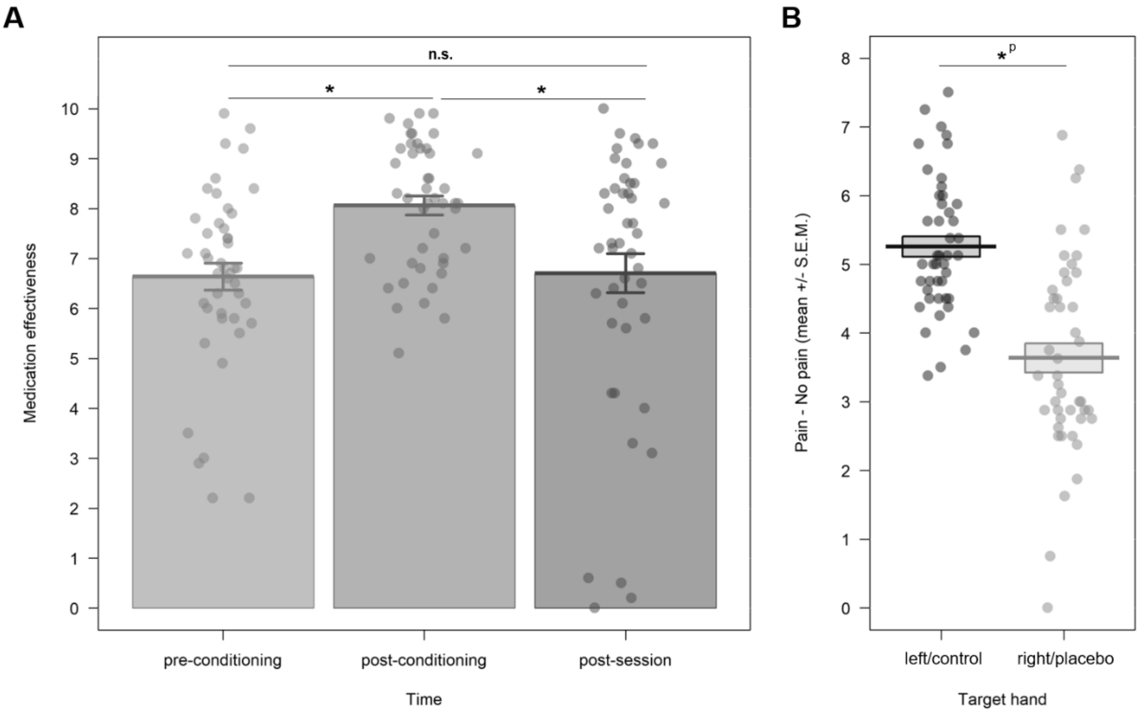

Figure S2. Behavioral manipulation checks to evaluate the strength of the first-hand placebo effect. Displayed are means and standard errors of the mean. A) We evaluated beliefs in the effectiveness of the administered gel at three times during the experiment - directly after the application by the medical cover (pre-conditioning), after the conditioning procedure (post-conditioning) and during the debriefing at the end of the session (post-session). This revealed a significant increase of beliefs after the conditioning. Although the beliefs significantly decreased until the end of the session, they did not drop significantly lower than initial belief levels, which were already high to begin with. B) We further evaluated first-hand pain ratings from another task done in the same session where individually calibrated, painful and non-painful electrical stimulation was delivered to the right and left dorsum of participant’s hands (for a thorough description of this task, see Hartmann et al., 2021); displayed here as an index of the ratings for painful – non-painful stimulation). Here we observed a significant placebo analgesia effect. * $p < .05$; S.E.M. = standard error of the mean; ^{p} = preregistered. Images taken from Figure 4 and Figure A1 in Hartmann et al. (2021).

R.3 Main behavioral analyses

Because the assumption of normality was violated for the pain rating data in the main study, we additionally calculated a Wilcoxon rank-sign test using the pain ratings. Similar to the preregistered ANOVA and *t*-test above, this did not reveal a statistically significant difference in pain ratings between the two hands (p = .293 one-tailed, Cohen's d = 0.03). Below we report the full ANOVA tables of behavioral results of the main study.

Table S2
Behavioral results of the ANOVA using pain ratings.

Effects	$F_{(1,44)}$	$p_{(two\text{-}tailed)}$	gen. η^2
target hand	5.42	.025	0.004
intensity	1348.88	< .001	0.93
target hand x intensity	0.14	.711	< 0.001

Table S3
Behavioral results of the ANOVA using unpleasantness ratings.

Effects	$F_{(1,44)}$	$p_{(two\text{-}tailed)}$	gen. η^2
target hand	15.56	< .001	0.004
intensity	327.89	< .001	0.74
target hand x intensity	2.04	.160	< 0.001

R.4 Study comparison

Here we report the full ANOVA tables of the exploratory comparison of pain and unpleasantness ratings between the validation and the main study.

Table S4
Behavioral results of the study comparison ANOVA using pain ratings.

Effects	$F_{(1,81)}$	$p_{(two\text{-}tailed)}$	gen. η^2
study	1.19	.277	0.01
target hand	4.00	.049	0.001
intensity	1649.51	< .001	0.91
study x target hand	2.57	.113	< 0.001
study x intensity	0.36	.545	0.002
target hand x intensity	< 0.001	.978	< 0.001
study x target hand x intensity	0.39	.528	< 0.001

Table S5
Behavioral results of the study comparison ANOVA using unpleasantness ratings.

Effects	$F_{(1,81)}$	$p_{(two\text{-}tailed)}$	*gen.* η^2
study	11.37	.001	0.73
target hand	11.78	< .001	0.002
intensity	576.74	< .001	0.75
study x target hand	3.09	.083	< 0.001
study x intensity	3.42	.068	0.02
target hand x intensity	0.75	.329	< 0.001
study x target hand x intensity	1.77	.188	< 0.001

R.5 Region of interest analyses

Below we report the full ANOVA tables of the fMRI results (pooled activation of all ROIs and single ANOVAs for each ROI) as well as the post hoc Bayesian analyses.

Table S6
ANOVA using pooled activation of seven ROIs.

Effects	*df*	*F*	$p_{(two\text{-}tailed)}$	*gen.* η^2
target hand	1,44	0.16	.689	< .001
intensity	1,44	13.21	< .001	0.016
roi^{S}	6,264	20.50	< .001	0.144
target hand x intensity	1,44	3.09	.086	0.002
target hand x roi^{S}	6,264	3.35	.003	0.002
intensity x roi^{S}	6,264	20.19	< .001	0.028
target hand x intensity x roi^{S}	6,264	4.61	< .001	0.002

Note. Effects marked with an “S” had a significant Mauchly's test for sphericity and corresponding *p*-values are reported using Greenhouse Geisser sphericity correction. The following regions of interest were included: l/rAI = left/right anterior insula, aMCC = anterior midcingulate cortex, l/rS1 = left/right primary somatosensory cortex, l/rS2 = left/right secondary somatosensory cortex.

Table S7
ANOVA of left anterior insula.

Effects	$F_{(1,44)}$	$p_{(two\text{-}tailed)}$	*gen.* η^2
target hand	1.21	.278	0.002
intensity	39.98	< .001	0.162
target hand x intensity	8.93	.005	0.015

Table S8

ANOVA of right anterior insula.

Effects	$F_{(1,44)}$	$p_{(two\text{-}tailed)}$	*gen.* η^2
target hand	0.42	.521	< .001
intensity	9.09	.004	0.040
target hand x intensity	6.47	.015	0.012

Table S9

ANOVA of anterior midcingulate cortex.

Effects	$F_{(1,44)}$	$p_{(two\text{-}tailed)}$	*gen.* η^2
target hand	0.23	.636	< 0.001
intensity	16.88	< .001	0.053
target hand x intensity	3.64	.063	0.003

Table S10

ANOVA of left primary somatosensory cortex.

Effects	$F_{(1,44)}$	$p_{(two\text{-}tailed)}$	*gen.* η^2
target hand	0.03	.862	< 0.001
intensity	3.85	.056	0.003
target hand x intensity	0.39	.534	< 0.001

Table S11

ANOVA of right primary somatosensory cortex.

Effects	$F_{(1,44)}$	$p_{(two\text{-}tailed)}$	*gen.* η^2
target hand	7.59	.008	0.007
intensity	1.10	.300	0.002
target hand x intensity	< 0.001	.988	< 0.001

Table S12

ANOVA of left secondary somatosensory cortex.

Effects	$F_{(1,44)}$	$p_{(two\text{-}tailed)}$	*gen.* η^2
target hand	0.26	.614	< 0.001
intensity	0.03	.853	< 0.001
target hand x intensity	0.21	.885	< 0.001

Table S13

ANOVA of right secondary somatosensory cortex.

Effects	$F_{(1,44)}$	$p_{(two\text{-}tailed)}$	*gen.* η^2
target hand	4.00	.052	0.005
intensity	5.29	.026	0.010
target hand x intensity	0.54	.468	< 0.001

Table S14

Overview of the results in the Bayesian t-tests for the seven ROIs.

Region of interest	MNI coordinates	Prior	BF_{01}	BF_{10}
aMCC	-2 23 40	Cauchy (0, .707)	0.92	1.09
lAI	-40 22 0	Cauchy (0, .707)	0.11	9.11
rAI	39 23 -4	Cauchy (0, .707)	0.28	3.55
lS1	-39 -30 51	Cauchy (0, .707)	9.20	0.11
rS1	36 -36 48	Cauchy (0, .707)	6.29	0.16
lS2	-39 -15 18	Cauchy (0, .707)	4.11	0.24
rS2	39 -15 18	Cauchy (0, .707)	3.17	0.32

Note. MNI coordinates are given as x, y and z; AI = anterior insula; aMCC = anterior midcingulate cortex; r/lS1 = right/left primary somatosensory cortex; r/l S2 = right/left secondary somatosensory cortex; BF_{01} = Bayes Factor evidence for null hypothesis (H_0 vs. H_1); BF_{10} = Bayes Factor evidence for alternative hypothesis (H_1 vs. H_0); $BF_{10} = 1/BF_{01}$. Bayesian *t*-tests were run in JASP, one-sided for $\text{activity}_{\text{right/placebo hand}} > \text{activity}_{\text{left/control hand}}$ (lAI, rAI, aMCC) or $\text{activity}_{\text{right/placebo hand}} < \text{activity}_{\text{left/control hand}}$ (lS1, rS1, lS2, rS2).

References

Benedetti F, Arduino C, Amanzio M. 1999. Somatotopic activation of opioid systems by target-directed expectations of analgesia. J Neurosci. 19(9):3639–3648.

Bingel U, Lorenz J, Schoell ED, Weiller C, Büchel C. 2006. Mechanisms of placebo analgesia: rACC recruitment of a subcortical antinociceptive network. Pain. 120(1–2):8–15.

Büsch D, Hagemann N, Bender N. 2009. Das Lateral Preference Inventory: Itemhomogenität der deutschen Version. Zeitschrift für Sport. 16(1):17–28.

Bynion TM, Feldner MT. 2017. Self-assessment manikin. In: Zeigler-Hill V, Shackelford T, editors. Encycl Personal Individ Differ. Cham: Springer.

Geuter S, Eippert F, Hindi Attar C, Büchel C. 2013. Cortical and subcortical responses to high and low effective placebo treatments. Neuroimage. 67:227–236.

Hartmann H, Rütgen M, Riva F, Lamm C. 2021. Another's pain in my brain: No evidence that placebo analgesia affects the sensory-discriminative component in empathy for pain. Neuroimage. 224:117397.

Jackson PL, Meltzoff AN, Decety J. 2005. How do we perceive the pain of others? A window into the neural processes involved in empathy. Neuroimage. 24(3):771–779.

Murray FS, Safferstone JF. 1970. Pain threshold and tolerance of right and left hands. J Comp Physiol Psychol. 71(1):83–86.

Oldfield R. 1971. The assessment and analysis of handedness: The Edinburgh Inventory. Neuropsychologia. 9:97–113.

Pud D, Golan Y, Pesta R. 2009. Hand dominancy - a feature affecting sensitivity to pain. Neurosci Lett. 467(3):237–240.

Rütgen M, Seidel E-M, Silani G, Riečanský I, Hummer A, Windischberger C, Petrovic P, Lamm C. 2015. Placebo analgesia and its opioidergic regulation suggest that empathy for pain is grounded in self pain. Proc Natl Acad Sci U S A. 112(41):E5638–E5646.

Schenk LA, Sprenger C, Geuter S, Büchel C. 2014. Expectation requires treatment to boost pain relief: An fMRI study. Pain. 155(1):150–157.

Tran US, Stieger S, Voracek M. 2014. Evidence for general right-, mixed-, and left-sidedness in self-reported handedness, footedness, eyedness, and earedness, and a primacy of footedness in a large-sample latent variable analysis. Neuropsychologia. 62(1):220–232.
